# An Account of the Effects of Labour in the Cure of Pulmonary Consumption. In a Letter from the Rev. Dr. Samuel K. Jennings, of Bedford County, Virginia, to Dr. Benjamin Rush

**Published:** 1807-11

**Authors:** Samuel K. Jennings


### *An Account of the Effects of* Labour *in the Cure of Pulmonary Consumption. In a Letter from the* Rev. Dr. Samuel K. Jennings, *of Bedford County, Virginia, to* Dr. Benjamin Rush.

Dear Sir,

THAT theory only is to be considered a rational one, which is supported by facts, and will admit of the most extensive practical utility. If the following facts can be of any service to you, it will afford me singular satisfaction to have communicated them.

I myself furnish the case. My maternal grandmother, my mother, five of her sisters, and four of her brothers, my sister being my mother's first child, and a brother next in succession to me by birth, all of them have been swept off the stage of life in the course of my recollection, by the fatal disease Phthisis Pulmonalis. From my youth up to the age of twenty-nine, I was sensible of great debility of the lungs, and was never, during that time, able to call aloud, read, or sing, with the ease which is common to other people. I had generally lived a studious and sedentary life, except that I had been the two last years engaged partially in the practice of physic. An offer was at that time made me to take charge of an academy. For the sake of gaining more leisure for the purpose of reading and study, I accepted the offer. In the mean time I had been three years occasionally employed in speaking upon religious subjects. From this last engagement I considered my lungs to have gained some strength. It followed, however, that study and confinement did less agree with me than formerly. I could perceive a daily declension; and at length, having been caught in a moderate rain, I was seized with a very severe and obstinate cough. I was bled again and again to no purpose. After considerable depletion, opium was tried, but in vain. Debility, the cough, and every inflammatory symptom increased. I had recourse to riding, took a journey of several weeks, and continued to let blood as often as the pains were severe, but still in vain. In the mean time I obtained your Inquiries, and immediately turned my attention to the subject which most concerned me. After having carefully read that part of the work, I pursued the following plan, viz. I let blood, moderately, every third day, especially if affected with with inflammatory symptoms, until, with the previous blood-lettings, I had been bled fifteen times in the course of five weeks. By this time I was much reduced, but my cough was no better. I then had recourse to the use of the AXE, and to LABOUR of the severest kind. I could not at the time repeat ten strokes without rest. It would seem in the first instance to increase my cough. The result was, that in two weeks I was nearly recovered. Finding much amendment, I grew remiss in my labour, and in a few weeks relapsed, and was nearly as ill as before, for I lost ground rapidly in the second instance. Two bleedings and similar LABOUR, however, finally restored me to good health, and I can now sing aloud, and on a sharp and high key; can speak two hours together; and, in one word, I consider myself freed from every symptom of that disorder.

My wife furnishes a second recent case. Her mother, and one of two only sisters, have died of the same disease very lately. She was in her youth an active and industrious woman, and of course took a good deal of laborious exercise. But for several years past she has been declining, so that, from a fleshy and healthy woman, she became a pale, sickly, emaciated, valetudinarian. The last summer she brought a fine son. By suckling him she declined in an unusual degree; was at length taken with a cough, chills at noon and in the evening, night sweats, &c. I bled her as often as I could find her pulse tense; advised her (contrary to her inclinations) to use SERVILE LABOUR. She took my advice. Her cough is nearly removed, and I have no doubt but she will recover,

I should not have considered these cases of sufficient importance to call your attention, had it not been for the hereditary circumstances attending them.

In my own case they are indeed striking, for not only the persons named above, but a number of my maternal cousins have died of the same disease.

I shall offer a short reflection or two, drawn from my own case. In the first place, I am persuaded that hard labour, if employed in an early stage, can cure the hereditary predisposition in some cases. Hence I further conclude, that consumptive parents ought never to choose sedentary or light employments for their children.

Secondly, I conclude, that although a trotting horse may afford sufficient exercise for many, yet labour will be far more successful.

And lastly, in all cases, the labour should be such as to require considerable efforts on the part of the patient. I laboured *continually*, and rarely with sufficient intervals to refresh myself by rest.

I am sincerely,

Sir, your most obedient,

SAMUEL K. JENNINGS.

*October 25th*, 1804.

